# Additive effects of eukaryotic co-expression plasmid carrying GRIM-19 and LKB1 genes on breast cancer *in vitro* and *in vivo*

**DOI:** 10.3892/mmr.2021.12226

**Published:** 2021-06-16

**Authors:** Wei Zhang, Ying Shao, Ye Du, Wei Geng, Tong Jiang, Haipeng Liu, Duo Zhang

Mol Med Rep 12: 7665-7672, 2015; DOI: 10.3892/mmr.2015.4393

Following the publication of this paper, it was drawn to the Editors’ attention by a concerned reader that the western blotting data featured in Figs. 1C and 4C, and tumour images in Fig. 5A, were strikingly similar to data appearing in different form in other articles by different authors at different research institutes. Owing to the fact that the contentious data in the above article were already under consideration for publication, or had already been published, elsewhere prior to its submission to Molecular Medicine Reports, the Editor has decided that this paper should be retracted from the Journal. The authors did not reply to indicate whether or not they agreed with the retraction of the paper. The Editor apologizes to the readership for any inconvenience caused.

